# Reducing Feature Size in Laser Implantation Texturing †

**DOI:** 10.3390/mi15080958

**Published:** 2024-07-27

**Authors:** Bart Ettema, Dave Matthews, Gert-Willem Römer

**Affiliations:** Faculty of Engineering Technology, University of Twente, Drienerloolaan 5, 7522 NB Enschede, The Netherlands; b.r.ettema@utwente.nl (B.E.); d.t.a.matthews@utwente.nl (D.M.)

**Keywords:** laser implantation, laser texturing, laser dispersion, surface texturing, surface structuring

## Abstract

Embossing rolls are used in a variety of sectors to transfer surface textures to a product. Textures on the rolls are typically achieved by material-removal techniques, resulting in craters in the surface of the roll. The wear resistance of the surfaces is improved by additional coating technologies. A novel process offering improved surface design freedom and which negates the need for post-coating techniques is the embedding of micro-meter-sized ceramic particles in the surface of the roll. This can be achieved through micro-additive processing. This work presents and discusses experimental results of surface texturing through locally derived laser-induced melt pools in which ceramic particles are dissolved. This process is termed laser implantation, or laser dispersing. Using this technology, dome-shaped surface structures with significantly increased hardness compared to the bare steel can be achieved. Reported results in the literature focus on implantations with diameters ranging from 150 μm to 400 μm and heights ranging from 10 μm to 30 μm. However, features with smaller diameters and heights are desired for technology adoption to permit a wider range of surface roughness. This paper presents and discusses the experimental results of implantations with a diameter smaller than 150 µm, with heights between 1 μm and 15 μm. For that purpose, a Nd:YAG laser source (focal diameter 70 μm, pulse durations from 3 to 15 ms, pulse power from 20 to 50 W average) was used to induce a melt pool driving the particle embedding.

## 1. Introduction

Textured metal sheets are typically finished through the process of skin pass rolling [1]. Here, the skin pass roll serves a dual function, namely both as a roller and a die, which transfers its surface pattern onto the surface of the metal sheet. The potential for variations in this surface texture on the sheet depends on how the inverse texture on the skin pass roll is produced. The longevity of the roll, as well as the quality of the resultant sheet texture, are directly impacted by the wear rate of the texture on the skin pass roll throughout a rolling sequence [2].

Current roll texturing techniques, such as shot blasting [3] and electrical discharge texturing (EDT) [4], involve removal and/or redistribution of material at the roll’s surface. The textures obtained through these techniques are often stochastic in nature. A common enhancement step in the production of skin pass rolls involves the application of hard chrome plating, increasing its wear resistance [5,6]. Due to environmental concerns, the use of chrome has been restricted in many industrial contexts [7].

In addition to wear-resistant rolls, there is a growing necessity for more design freedom of the roll’s surface texture and inturn of the metal strip. The stochastic texturing techniques discussed above do not offer this design freedom. Research by Gorbunov et al. [3] evaluated five texturing methodologies, which generate textures that can be perceived as (pseudo-)deterministic on the skin pass rolls. These methods predominantly depend on removal or material redistribution at the microscopic scale. The authors concluded that additive material techniques for texture creation are promising for rolls in steel sheet production. Additive manufacturing not only promises greater design flexibility, but might also enhance the texture’s durability and therefore the roll’s lifespan.

The additive manufacturing technique termed “laser implantation texturing” (LITex) [8], allows for strategically depositing a deterministic texture on a substrate, characterized by a sequence of “implants”. Another term commonly associated with this technique is “laser dispersing” [9]. By specifically selecting the material to be dispersed, the hardness of the surface texture of the roll can be improved, potentially eliminating the need for a chrome coating.

**Figure 1:**
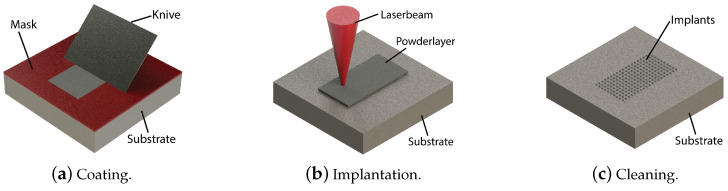
Schematic representation of the various steps in the laser implantation process: (**a**) application of a powder layer, (**b**) application of a powder layer and (**c**) removal of excess powder.

The LITex process unfolds in three steps, illustrated schematically in Figure 1. During the first step (Figure 1b), a layer, with a thickness of about 100 μm, is deposited onto the substrate. Typically, the layer comprises a compound of micro-meter-sized ceramic particles suspended in a binder (for example polyvinylbutyral (PVB)). The layer is applied using, for example, a mask with the intended layer thickness and spread with a knife edge [10]. For the implantation to be wear resistant, the selected particles must exhibit a hardness higher than the substrate and have a melting point which is higher than that of the tool steel (substrate). Concurrently, the chosen particles should exhibit a minimal thermal expansion coefficient to prevent stress formation during solidification. To ensure the success of the implantation, particle diameters must be notably less than the desired implant diameter. In the subsequent step (Figure 1), the powder layer is exposed to a pulsed laser beam, which causes the binder to evaporate and the substrate to melt, enabling the particles to submerge (sink) into the melt pool. Following the solidification of the melt pool after the laser pulse, the particles are anchored within the substrate, creating a protruding “implant”. In the final step (Figure 1c), a cleaning process removes excess slurry surrounding the implants. This implantation routine can be repeated, either by repositioning the laser beam or adjusting the substrate’s position, to generate patterns of implants. Frequently used abbreviations are listed at the end of this paper.

Hilgenberg and Steinhof [8,11] developed this methodology to successfully produce implant diameters spanning from 150 μm to 400 μm and implant height between 10 μm and 30 μm. Their findings, achieved using laser powers ranging from 40 W to 180 W, a focal spot diameter of 105 μm, pulse durations between 3 ms and 15 ms, and a 100 μm-thick powder layer composed of ceramic particles sized between 5 μm and 13 μm, show a texture hardness of up to 1800 Hv when applied to an AISI D2 substrate.

In industrial contexts, particularly for skin rolls, there is a pressing need for implantation diameters smaller than 150 μm [3], i.e., smaller than the implants achieved in [8,11]. In recent years, advances in laser implantation have been reported [12,13]. However, these studies focus the application of laser-implanted textures on stamping tools, instead of aiming to decrease the size of the laser-implanted textures. Decreasing the feature size in LITex will enable a greater degree of surface design freedom, as well as making the process viable to a wider range of engineering sectors. The research detailed in this article presents and discusses efforts to achieve reduced implantation diameters without compromising the enhanced hardness imparted by ceramic particles. To that end, we established a laser process window for one type of ceramic particles (WC), including factors such as layer thickness and spot size, among others, as well as an assessment of the hardness of the smaller implants.

## 2. Methodologies

### 2.1. Experimental Setup

**Figure 2:**
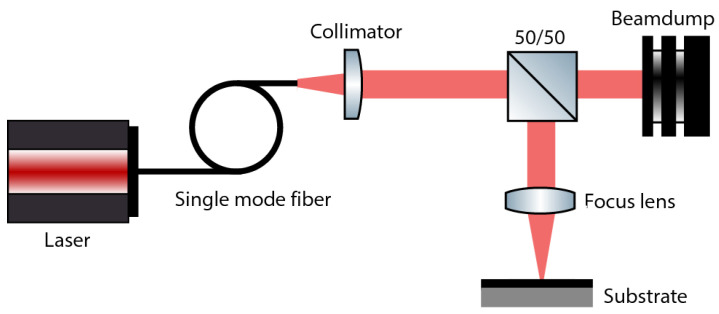
Schematic representation laser implantation setup.

A schematic representation of the experimental setup is shown in Figure 2. This setup incorporates an Nd:YAG fiber laser source (JK100FL of JK Lasers, West Bromwich UK). This laser source emits a beam with a wavelength of 1080 nm. The emitted beam is transported through a single-mode optical fiber, into a collimator with a focal length of 76 mm.

A non-polarizing 50:50 beam splitter (CCM1-BS014/M from Thorlabs Inc., Newton, NJ, USA) was integrated in the beam path. The splitter is combined with a beam dump to enable the laser source to operate efficiently and stable at elevated power levels while modulating the emitted power to the power levels needed for the process. Following the beam splitter, a focus lens (provided by JK Lasers, West Bromwich, UK) with a focal length of 300 mm is utilized to focus the laser beam to a diameter of 70 μm. The latter diameter was measured using a MicroSpotMonitor (MSM+ of Primes GmbH Pfungstadt, Pfungstadt, Germany). From the caustic measurements using this tool, a beam quality of $M^{2}$ = 1.23 was found. Additionally, to inhibit oxidation during laser processing, argon is supplied at a rate of 10 L/min via a nozzle (inner diameter of 6 mm) situated at about 20 mm to the laser material interaction zone, at an angle of about 45 degrees to the normal on the substrate surface. The sample is positioned relative to the stationary beam using an XY stage consisting of two DDS100/M (Thorlabs Inc., Newton, NJ, USA). The repeatability accuracy of these stages are ±1.5 μm. During the experiments, laser pulse powers were varied between 20 W and 50 W using pulse durations ranging from 3 ms to 20 ms. Processing parameters are presented and discussed in Section 2.5.

### 2.2. Materials

The substrate utilized comprised slabs of X100CrMoV5 (procured from Udeholm, Amsterdam, The Netherlands) tool steel, each measuring 30 × 30 mm^2^ and having a thickness of 6 mm. The chemical composition of this steel is presented in Table 1. X100CrMoV5 is a common choice of tool steel for skin pass rolls, due to the chromium carbides within this ledeburitic steel. These carbides result in superior wear resistance.

The substrate’s surface underwent a polishing procedure using sandpaper with decreasing grit sizes to achieve a final roughness value of Ra = 0.2 µm. Subsequent heat treatments, including vacuum hardening and annealing, were applied to the samples, culminating in a hardness measure of 600 Hv0.2 ± 30. Other material properties are listed in Table 2.

### 2.3. Powder Particles

The chosen particles to be implanted were tungsten carbide (WC) and titanium diboride (TiB_2_), with average diameters below 2 μm and 10 μm, procured from Sigma-Aldrich (Saint Louis USA), because of their high hardness and melting point. The material properties as provided by the manufacturer are summarized in Table 3.

**Figure 3:**
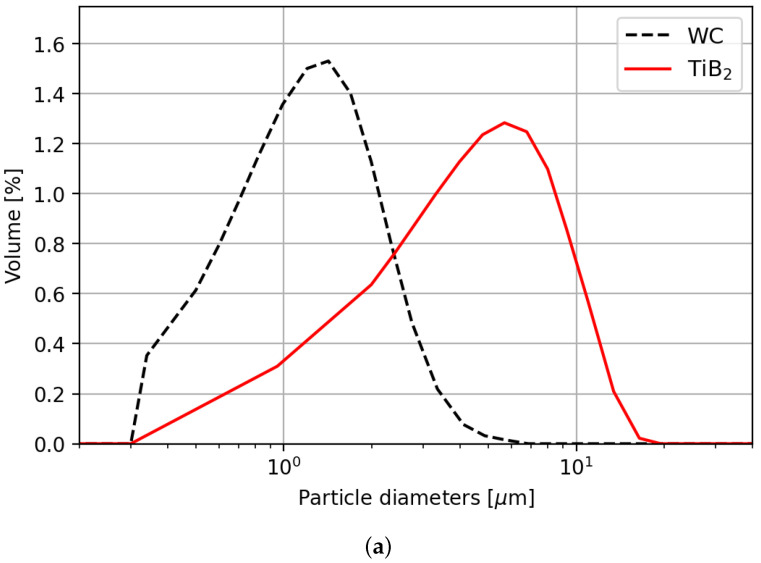

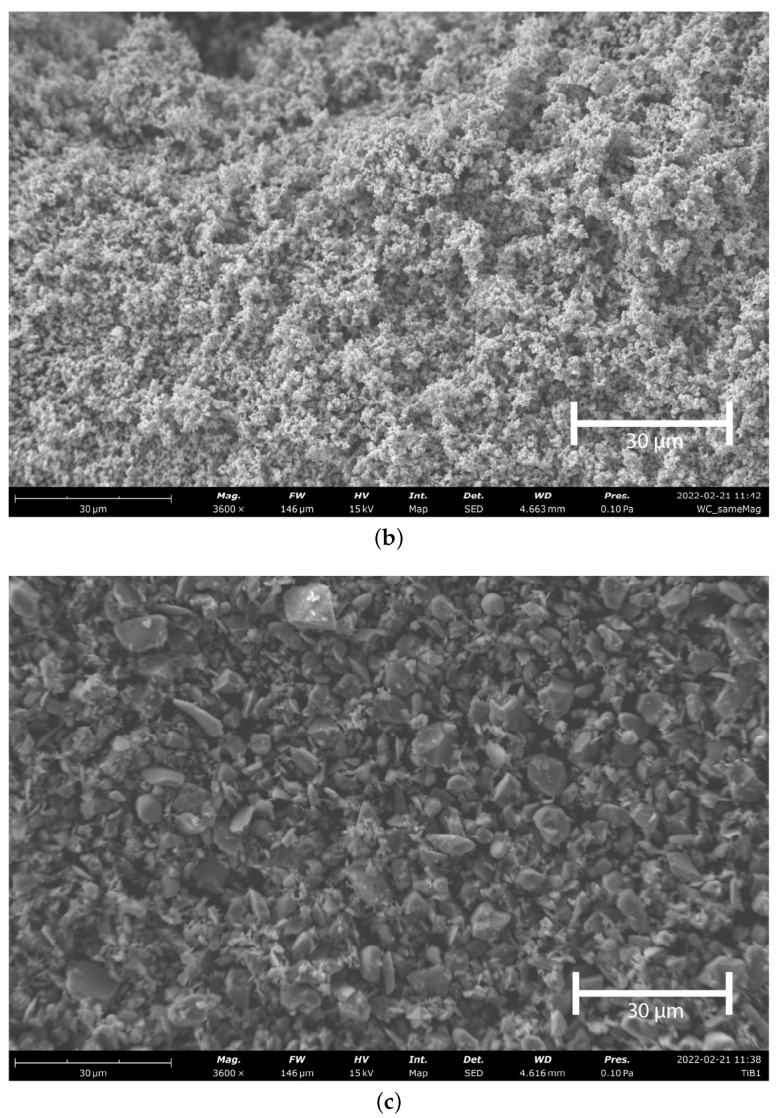
Characteristics of ceramic particles. (**a**) Particle-size distribution of WC and TiB_2_ powder (measured with Sympatec HELOS (H4766) and RODOS/T4, R2+R4 l). (**b**) SEM micrograph of WC. (**c**) SEM micrograph of TiB_2_.

However, laser diffraction measurements of the particles, using a Sympatec HELOS H4766 (form Sympatec Clausthal-Zellerfeld Germany), show a significant deviation of the average specified diameters see (Figure 3a). Also, SEM imaging Figure 3b shows the conglomerating nature of the small WC particles, which is common for micro-meter-sized particles [20] and decreases the reliability of depositing layers of uniform thickness on the substrate. Furthermore, SEM imaging of the TiB_2_ particles shows sharp and fractured features, but shows less conglomerations due to their larger average diameters of about 4.23 μm (see Figure 3c).

As an adhesive agent to bind the particles, polyvinyl butyral (PVB, Mowital 30H procured from Kuraray, Tokyo, Japan), combined with an ethanol solvent, was employed to yield a paste mixed by hand-stirring in an erlenmeyer consisting of 18.6 wt.% ethanol, 77.5 wt.% particles, and 3.9 wt.% PVB. The evaporation temperature of the PVB is several magnitudes lower than the substrate’s melting temperature, meaning that the binder fully evaporates during the laser material interaction. This paste was applied on the substrate, using a mask and a knife-edge technique to ensure a layer of uniform thickness as mentioned in the introduction, Figure 1b. Masks with thickness of 50 μm and 75 μm, respectively, were used. After air drying for six hours, the ethanol solvent is fully evaporated, resulting in a uniformly distributed layer of 94 wt.% particles and 6 wt.% PVB. Subsequently, powder layers of 50 and 75 μm were formed on the substrates, with measured deviations not exceeding 10 % of the layer thickness.

### 2.4. Analysis Tools

To evaluate the geometric characteristics of the implants, a scanning electron microscope (JSM-7200F, JEOL, Tokyo, Japan) was utilized. Subsequent EDX imaging, facilitated by a sensor from Oxford instruments (Abingdon, UK), was conducted to characterize the spatial distribution and concentration particles elements within these implants.

An average hardness was determined by performing macro indentation using a hardness tester, Leco Inc., (Sint Joseph, MI, USA) LM 100, employing an indentation force of 200 gf. This indentation force results in indention diameters between 17 μm and 30 μm. Therefore, the indentation size is small compared to the implantation diameters and significantly larger that the particle size of the ceramic particles. The macro indentations were preformed in the center of the implants as a flat surface is required for accurate hardness measurements.

**Figure 4:**
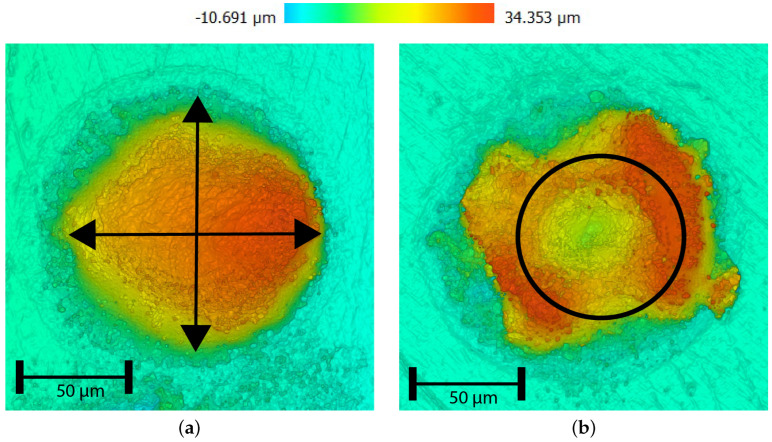
Paths used for height determination of implants using confocal microscopy images. (**a**) Average height determined along cross paths for dome-shaped implants. (**b**) Average height determined along circle path for ring shaped implants.

Confocal microscopy images were made (S neox, Sensofar Tech S.L., Barcelona, Spain) for determination of the implant’s dimensions, specifically its diameter and (average) height. For implants exhibiting an elliptical shape, the diameter $D_{i}$ was determined by averaging the lengths of both the major and minor axes (see Figure 4a). Analogously, an implant’s height $H_{i}$ was deduced by averaging the peak heights observed along these two axes for the dome-shaped implants. The heights of the ring-shaped implants are determined by averaging over the circle drawn on top of the ring, as shown in Figure 4b.

**Figure 5:**
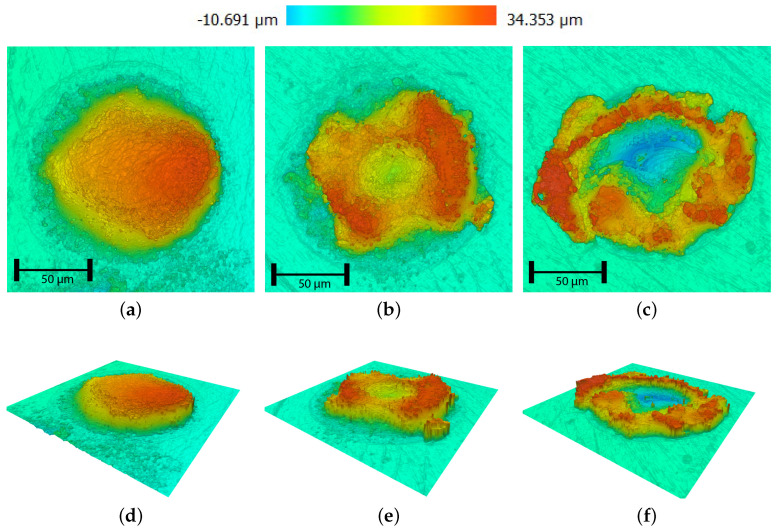
Confocal microscopy images of WC implants, $H_{l}$ = 75 μm, pulse time t_*p*_ = 6 ms. (**a**) Dome shape obtained at 20 W. (**b**) Ring shape obtained at 40 W. (**c**) Failed obtained at 60 W. (**d**) Dome shape obtained at 20 W. (**e**) Ring shape obtained at 40 W. (**f**) Failed obtained at 60 W.

A distinction is made between dome-shaped implants (Figure 5a) and ring-shaped implants (Figure 5b), as both dome- and ring-shaped implants are considered relevant for surface-texturing patterns. As reported in the literature, ring-shaped implantation geometry forms at increased laser intensity [21]. Keyhole effects and failed implantation occur for higher laser intensities (Figure 5c).

### 2.5. Methods

After preliminary experiments in which the laser power, pulse time, and powder layer thickness were varied, an experimental processing window was found. It was found that a decreased powder layer thickness is found to be pivotal in creating smaller implantations. Within this processing window, dome, ring, and failed implants could be created with a sub-150-μm diameter. Therefore, in this experimental study, the laser power during each pulse was adjusted to range between 20 and 50 W (see Table 4). This table also shows the range of corresponding average laser intensity levels from the laser power in the pulse and the focal spot size of 70 μm. The laser pulse duration was varied within a range of 3 ms to 20 ms. As mentioned, two powder layer thicknesses of 50 μm and 75 μm for both WC and TiB_2_ were employed. For every set of processing parameters, nine experiments were conducted to evaluate the reproducability of the implantation process. That is, for each set of processing parameters, both the mean dimensions and standard deviations of the implants were determined based on nine experiments.

## 3. Results and Discussion

### 3.1. TiB_2_ Implantations

**Figure 6:**
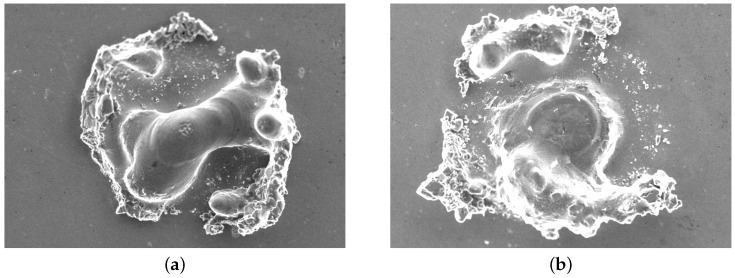
SEM micrographs of TiB_2_ implants after the cleaning step, $t_{p}$ = 3 ms, pulse power = 30 W. (**a**) Implant using $H_{l}$ = 75 μm. (**b**) Implant using $H_{l}$ = 50 μm.

Implantation using relatively large TiB_2_ particles at intensities ranging from 500 to 1300 kW/m^2^ and a spot radius of 70 μm fails to reliably generate dome- or ring-shaped implants, as illustrated by Figure 3c. The latter is attributed to the ratio of the particles to the layer height being too large, hindering the mixing and implantation of the TiB_2_ particles. In turn, this leads to poor reproducibility of implantation geometries. Additionally, in the case of the 50 μm powder layer, keyholing occurs in the center of the implantation zone (see Figure 6b). When using TiB_2_ particles, none of the laser-processing parameters resulted in reproducible dome or ring-shaped implantations. Therefore this powder is excluded from further analysis in this study.

### 3.2. WC Implantations

**Figure 7:**
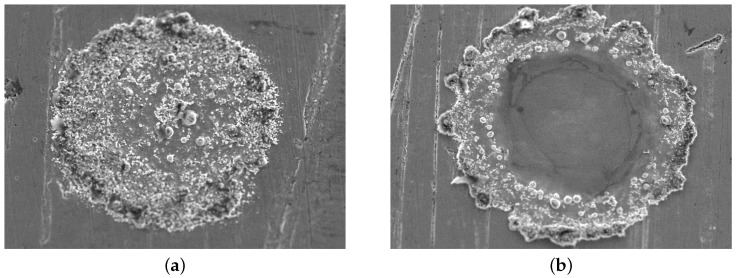
SEM micrographs of WC implants, layer height $H_{l}$ = 50 μm and pulse time $t_{p}$ = 3 ms. (**a**) Pulse power = 20 W. (**b**) Pulse power = 50 W.

Figure 7 shows SEM micrographs of both dome- and ring-shaped WC implants. In these images, the sharp WC particles can be observed at the surface of the implants. Also, the figure shows a typical dome-shaped implant (Figure 7a) and a ring-shaped implant (Figure 7b). The latter forms typically increased at laser intensities.

**Figure 8:**
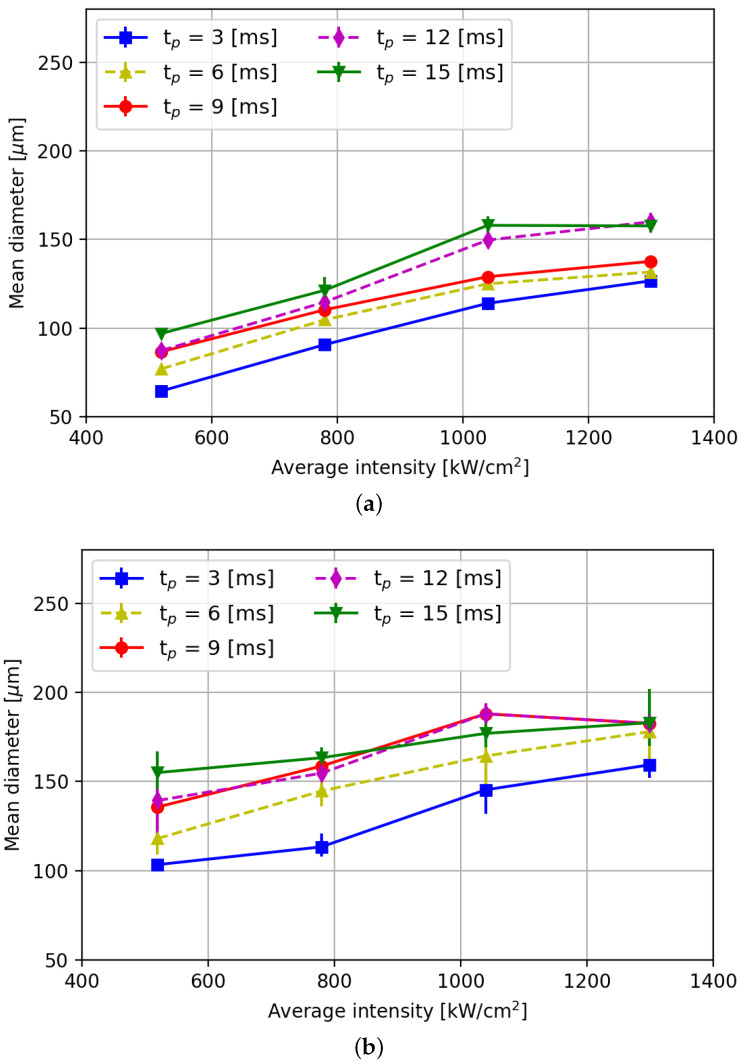
Measured mean diameters of WC implants as function of average laser intensity. (**a**) Layer height $H_{L}$ = 50 μm. (**b**) Layer height $H_{L}$ = 75 μm.

**Figure 9:**
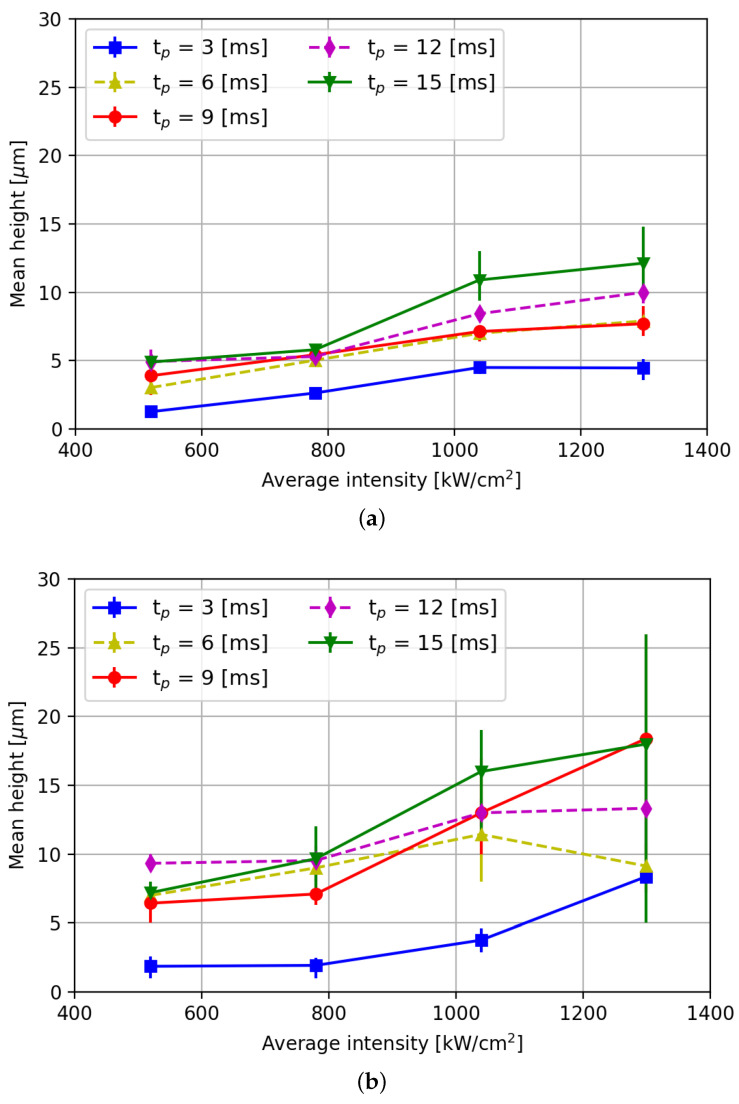
Measured mean height of WC implants as function of average laser intensity. (**a**) Layer height $H_{L}$ = 50 μm. (**b**) Layer height $H_{L}$ = 75 μm.

Figure 8 shows the corresponding averaged diameters and Figure 9 shows the height of implantations as function of the laser intensity. As can be observed from Figure 8a, the implantation diameter decreases with decreasing laser pulse power. It can also be concluded from this graph that decreasing the pulse duration leads to a decrease in the implantation diameter. The latter can be attributed to the lower total energy over time that the melt pool is exposed to. Furthermore, the geometry of the implantation is dome-shaped (Figure 7a) for average intensities up to 780 kW/cm^2^ and ring-shaped at 1040 kW/cm^2^ and higher (Figure 7b).

As can be concluded from the error bars in Figure 9, the variability in the height values of implants is found to be larger than the variability in implant diameter (Figure 8), particularly when using a pulse duration of 15 ms. For laser intensities of 780 kW/cm^2^ and below, the variation in implant height is comparatively smaller than for higher laser intensities across all pulse durations. Moreover, the difference in height of implants formed when using pulse durations between 3 ms and 6 ms is more pronounced than that between 6 ms and 12 ms. It is evident from the graph that reducing the pulse duration leads to a decrease in implant height. This phenomenon can be attributed to lower temperatures in the melt pool, resulting in increased viscosity and reduced surface tension in and on the melt pool [22,23].

It is noteworthy that at a pulse duration of 15 ms and an intensity of 1040 kW/cm^2^, the achieved implant height closely aligns with height values reported in the literature [8,11,24,25]. However, when using this parameter combination, the minimum implant diameter (Figure 8a) is smaller than the diameters reported in the literature. Therefore, by reducing laser intensity, powder layer thickness, and pulse duration, it is possible not only to achieve smaller implants, but also possible to create implants with an aspect ratio where the diameter is several times larger then the height. The latter is desirable to avoid large forces exerted on the implants that could lead to implant break-off during rolling. It is essential to note that smaller implants are only obtained when using a layer height of 50 μm and not when using 75 μm layers. Figure 8b and Figure 9b reveal that thicker layer heights (75 μm) yield implantation dimensions similar to those reported in the literature, in which a 100 μm layer height and a 105 μm spot diameter were reported [25]. This suggests that layer height significantly influences implantation diameter. All in all, it can be concluded from this graph that significantly smaller implant diameter than reported in literature can be reliably achieved, provided a low(er) layer thickness and a smaller spot diameter (here $h_{l}$ = 50 and 75 μm, $F_{s}$ = 70 μm) than typically applied ($h_{l}$ = 150–400 μm, $F_{s}$ = 105 μm [26,27]) are chosen. Similarly, the average height of the implants also shows a decreasing trend when decreasing the laser power, as can be concluded from Figure 9a.

### 3.3. Dispersion of WC Particles

**Figure 10:**
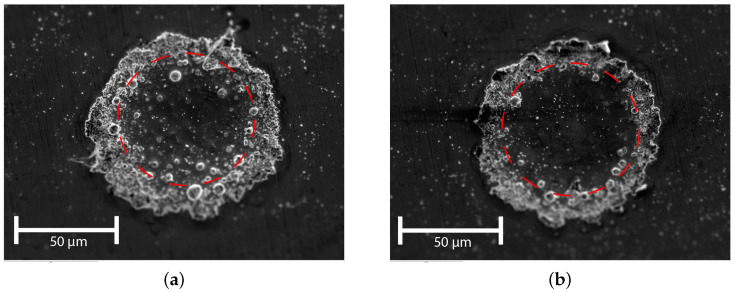
SEM micrograph (top view) showing the diameters of WC implants (pulse power = 30 W and layer $H_{L}$ = 50 μm) to different pulse durations. The red dotted circle indicates the laser spot. (**a**) $t_{p}$ = 3 ms, dome shaped. (**b**) $t_{p}$ = 9 ms, ring shaped.

Figure 10 shows an SEM micrograph of a typical WC implant, after the cleaning step. In this figure, the (size of the) laser spot is visualized by a red dotted circle. In this SEM image, the implant shows an outer diameter larger than the laser spot diameter, characterized by the light gray perimeter outside the laser spot. Hence, it can be concluded that the diameter of the melt pool during the implantation process is larger than the diameter of the laser spot. This is attributed to either conduction or the shoulders of the laser spot or a combination of both.

**Figure 11:**
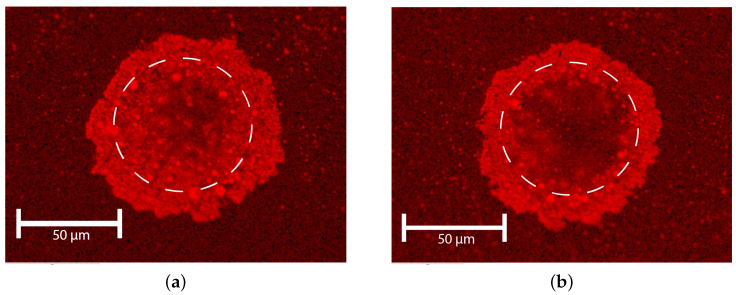
EDX micrograph (top view) showing tungsten concentration at the surface pulse power = 30 W. Same implants as Figure 10. (**a**) $t_{p}$ = 3 ms, dome shaped. (**b**) $t_{p}$ = 9 ms, ring shaped.

Figure 11 shows the EDX analysis of the WC implants, clearly showing tungsten concentration, especially in the perimeter of the implant. From this EDX analysis, it can also be concluded that the diameter of the melt pool was larger than the diameter of the laser spot. The EDX image suggests that in the ring-shaped implant, WC is mainly found in the perimeter of the implant, and not or at least in a reduced concentration in the center of the implant. The latter can be attributed to the increased energy in the ring-shaped melt pool, leading to melting of the WC particles.

**Figure 12:**
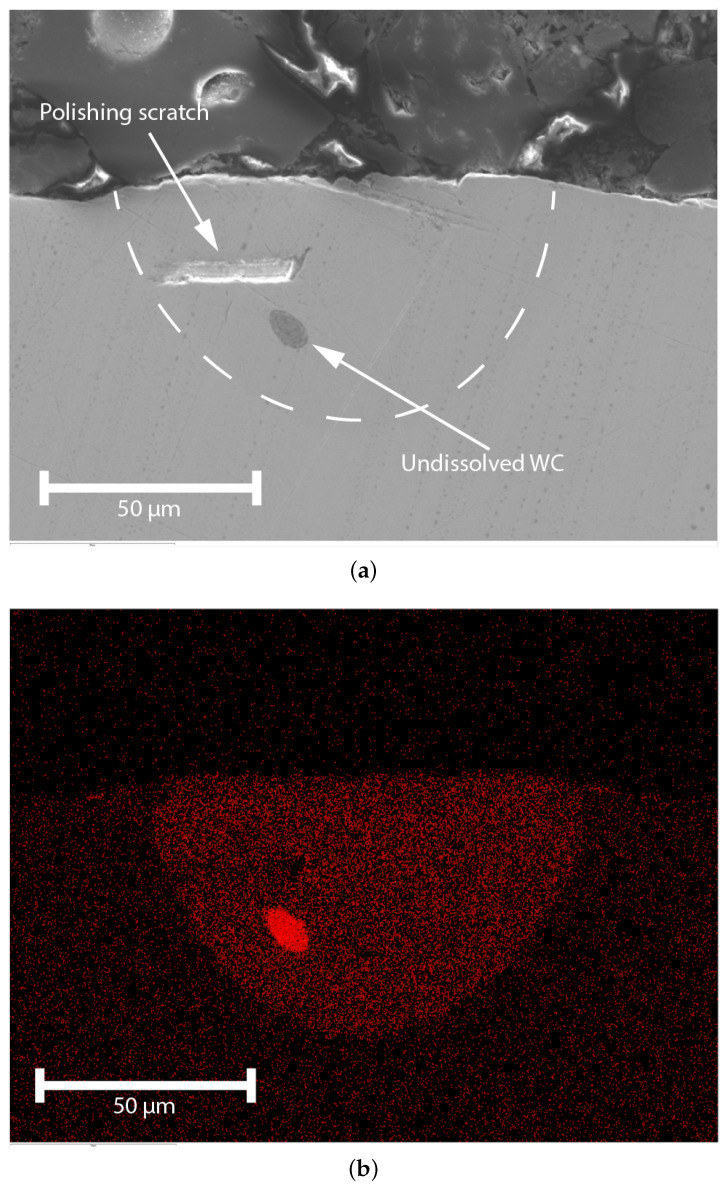
SEM micrograph and EDX cross-section of a dome-shaped implant, pulse power = 30 W, $t_{p}$ = 3 ms, and layer $H_{L}$ = 50 μm. (**a**) SEM micrograph cross-section, the white dotted line indicates the perimeter of the LIZ. (**b**) EDX micrograph cross-section.

Additionally, a cross-section EDX micrograph (Figure 11b) reveals increased Tungsten concentration caused by the homogeneously dissolved WC particles in the laser-induced melt pool. High concentrations of tungsten are found in the LIZ because WC particles do not fully dissolve. A single large “lump” of WC can be observed in Figure 12a,b. This lump might be caused by a large WC particle which did not fully dissolve, or by conglomeration of smaller particles.

The results per element of the cross-section shown in Figure 12, are presented in Table 5. The results reveal the significant presence of tungsten in the implants.

### 3.4. Hardness WC Implants

**Figure 13:**
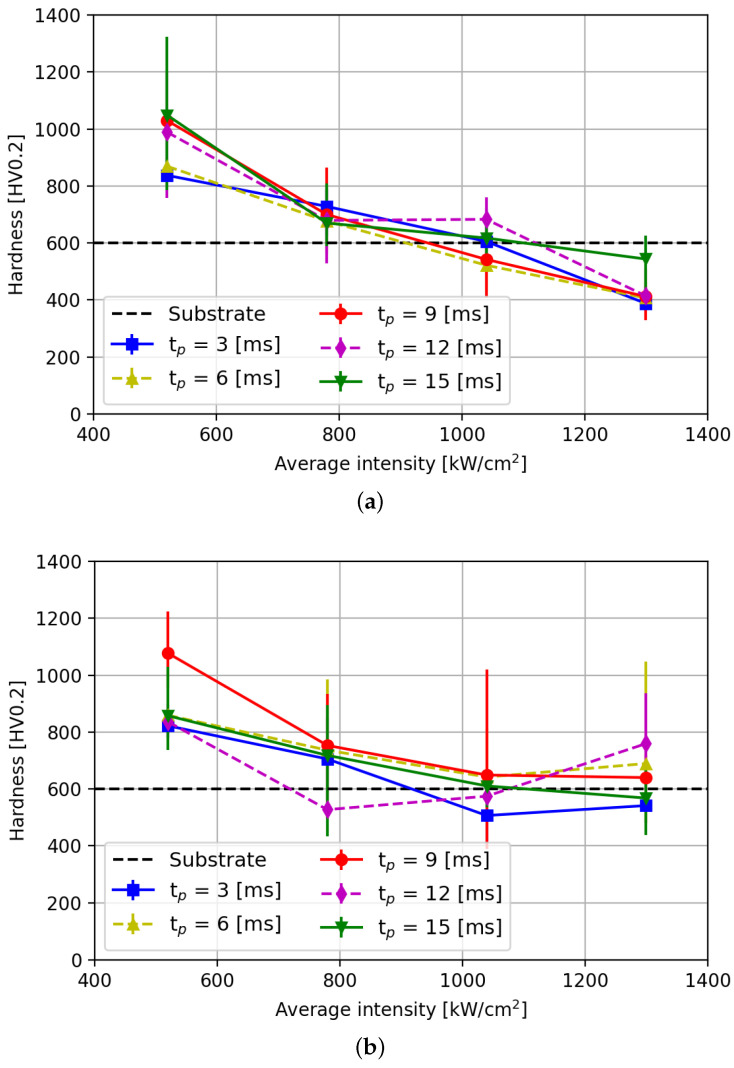
Measured micro-hardness of implants as function of the average laser intensity. The black dashed line indicates the hardness of the bare substrate. (**a**) Layer height $H_{L}$ = 50 μm. (**b**) Layer height $H_{L}$ = 75 μm.

Before implantation, the hardness of the bare substrate was measured and found to equal 600 Hv0.2 (see Table 2). Next, the hardness of the laser–material interaction zone of the substrate was measured. That is, the bare surface, without a powder layer, was melted by exposing it to a laser pulse power of 20 W and a pulse duration of 9 ms using the same spot diameter of 70 μm. After resolidification, the average hardness of the resulting laser–material interaction zone was found to equal 690 Hv0.2, which is harder than the hardness of the as-received substrate. The increased hardness is due to rapid laser solidification after the laser pulse [28]. Therefore, any hardness in WC implants exceeding 690 Hv0.2 can be attributed to the implantation of WC particles. Figure 13 shows the average hardness measured in the center of the implants using the Leco LM 100.

The average hardness of the implants as high as 1154 Hv0.2 can be achieved with a laser intensity of 500 kW/cm^2^ and pulse duration of 9 ms or longer. The latter is similar to the hardness values reported by Spranger [26], but with decreased implantation dimensions. As can be concluded from the increasing intensity, the hardness in the center of the implants drops. The latter is attributed to a lower concentration of dissolved WC particles in the center of the implants. The tungsten concentration is the main contribution to the increased hardness of the micro-structure within the implantations [29].

Implants created at laser intensities above 1040 kW/cm^2^ show a similar or lower hardness than the substrate hardness. This is attributed to the fact that hardness is measured in the center of the implants; and ring-shaped implants have a lower tungsten concentration in their center (see Figure 11b). These centers yield lower hardness than the substrate hardness; this is attributed to annealing of the material and absence of dissolved tungsten [30]. The hardness of the outer diameter is expected to yield similar results to the hardness of implants forming at lower intensity. However, accurate hardness measurements are unfeasible due to the lack of a significant flat surface to preform indentations on.

## 4. Conclusions

Experimental results of laser dispersing or laser implantation texturing (LITex) using tungsten carbide (WC) particles (d50 = 1.15 μm) in a 50 μm powder layer on a tool steel substrate and a Nd:YAG laser (pulse power 20 W to 50 W, pulse duration, 3 ms to 15 ms, focal diameter 70 μm) were analyzed. Dimensional analysis of the implants showed implant diameters ranging from 64 μm to 159 μm, with heights ranging from 1 μm to 13 μm. Also from the analysis, the following conclusions are drawn:

- a laser spot intensity of 500 kW/cm^2^ creates dome-shaped implants,
- a laser spot intensity above 500 kW/cm^2^ yields ring-shaped implants,
- decreasing the laser pulse power decreases the dimensions of the implants,
- increasing the laser pulse time increases the dimensions of the implants,
- and implantation diameters as small as 64 μm and heights as small as 1 μm could be produced.

However, the reproducibility of the LITex process reduces if laser pulse powers of more than 30 W are applied. The observed diameters of implants obtained at less than 30 W are smaller than implant dimensions reported in literature. These smaller dimensions are essential for the applicability of LITex as a laser patterning technique in the texturing of skin pass rolls in the steel industry. Micro-hardness measurements of the implants show an average hardness as high as 1152 Hv0.2, which is well above the hardness of 600 Hv0.2 of the virgin tool steel substrate. The higher hardness of the implants is expected contribute to the wear resistance of the steel surface.

## Figures and Tables

**Table 1:** Chemical composition substrate material in wt.% [14].

C	Si	Mn	Cr	Mo	V
1%	0.3%	0.16%	5.3%	1.1%	0.2%

**Table 2:** Material properties substrate [14,15].

	X100CrMoV5
Density [kg/m^3^]	7860
Melting point [°C]	1424
Hardness [Hv]	600

**Table 3:** Properties of ceramic powders [16,17,18] and binder [19].

	WC	TiB_2_	Mowital 30H
Specified particle size d50 [μm]	2	10	-
Measured d50 [μm]	1.15	4.23	-
Density [kg/m^3^]	1560	4520	1080
Hardness [Hv]	2200	3400	-
Melting point [°C]	2785	3230	165

**Table 4:** Experimental laser-processing parameters.

	WC	TiB_2_
Layer height [μm]	50, 75	50, 75
Laser pulse duration [ms]	3–20	3–20
Laser pulse power [W]	20–50	20–50
Laser spot diameter [μm]	70	70
Average laser intensity [kW/cm^2^]	520–1300	520–1300

**Table 5:** Quantitative analysis of EDX cross-section in Figure 12b.

Element	Fe	W	C	Cr	Mo	Si	V	Ca
wt.%	75.6	9.2	9.1	4.6	0.9	0.3	0.2	0.1

## Data Availability

The original contributions presented in the study are included in the article, further inquiries can be directed to the corresponding author.

## Abbreviations

The following abbreviations are used in this manuscript:

LITex	Laser implantation texturing
SBT	Shot blast texturing
EDT	Electron discharge texturing
ECD	Electro-chemical deposition
EBT	Electron beam texturing
LT	Laser texturing
EDX	Energy dispersive X-Ray
HAZ	Heat-effected zone
LIZ	Laser-implanted zone
SEM	Scanning electron microscopy
